# Supplementary material for: Association of healthcare fragmentation with three-year survival among kidney transplant recipients in Colombia
Source: PLoS One. 2025 Aug 18;20(8):e0316418. doi: 10.1371/journal.pone.0316418 (PMC12360509; doi:10.1371/journal.pone.0316418)
Supplement: S1 Text — (PDF) [file pone.0316418.s001.pdf]

La salud es de todos | Minsalud

201913000267461

Radicado No.: **201913000267461**
Fecha: **05-03-2019**

Bogotá D.C.,

Doctor
GIANCARLO BUITRAGO
Profesor asociado Departamento de Cirugía
Instituto de Investigaciones Clínicas
Universidad Nacional de Colombia
gbuitragog@unal.edu.co
No registra
Bogotá D.C.

ASUNTO: Solicitud información salud, Rad.201842301411402

Cordial saludo.

En atención a su solicitud de información anonimizada para las fuentes de interés, nos permitimos informarle:

1. Para el conjunto de datos de: Afiliados a salud, PILA, Información de Servicios de Salud – Estudio de Suficiencia y Nacimientos/Defunciones – RUAF-ND del período 2010-2016, se autoriza al Departamento de Epidemiología Clínica y Bioestadística a realizar la entrega de este conjunto de datos (adjunto oficio de autorización, rad.201913000267381) al Instituto de Investigaciones Clínicas de la Universidad Nacional.

2. Para el conjunto de datos de MIPRES, es necesario que definan las variables de interés, para lo cual se sugiere revisar la Resolución 1885 de 2018.

3. El conjunto de datos de Afiliados a salud, PILA e Información de Servicios de Salud – Estudio de Suficiencia del año 2017 y RIPS 2011-2017 están siendo procesados y se realizarán entregas graduales, debido al tiempo de procesamiento que demandan y volumen que ocupan. En la medida que se tengan disponibles los archivos se les informará para que dispongan del dispositivo externo para su respectiva copia.

Se hace entrega de esta información en los términos de la Ley 1581 de 2012 – *por el cual se dictan disposiciones generales para la protección de datos personales* – y su uso debe garantizar el derecho de habeas data. Es importante tener en cuenta la Corte Constitucional en Sentencia C-748/2011, señala que una vez la entidad administrativa

accede al dato personal adopta la posición jurídica de usuario dentro del proceso de administración de datos personales, lo que de forma lógica le impone el deber de garantizar los derechos fundamentales del titular de la información, previstos en la Constitución Política y en consecuencia deberán:

- *(i) " guardar reserva de la información que les sea suministradora por los operadores y utilizarla únicamente para los fines que justificaron la entrega, esto es, aquellos relacionados con la competencia funcional específica que motivó la solicitud de suministro del dato personal;*
- *(ii) informar a los titulares del dato el uso que le esté dando al mismo;*
- *(iii) conservar con las debidas seguridades la información recibida para impedir su deterioro, pérdida, alternación, uso no autorizado o fraudulento; y*
- *(iv) cumplir con las instrucciones que imparta la autoridad de control, en relación con el cumplimiento de la legislación estatutaria"*.

Por parte de esta Oficina se solicita socializar la existencia de estos datos al interior de la Universidad, en aras a facilitar su consulta por parte de los interesados y evitar reprocesos a nivel del Ministerio de esta misma información, optimizando de esta manera los recursos.

Finalmente, es importante resaltar que para el Ministerio de Salud y Protección Social y en particular para esta Oficina será de gran beneficio conocer los resultados de los proyectos de investigación que ustedes adelanten utilizando los datos entregados, por lo que estaremos atentos a conocer el resultado de esos proyectos.

Atentamente,

DOLLY ESPERANZA OVALLE CARRANZA
Jefe Oficina de Tecnología de la información y la Comunicación

Adjunto: Oficio autorización entrega Universidad Pontificia Universidad Javeriana

Elaboró: LuzR / Revisó/Aprobó: MaríaE
C:\LRincon\Trabajo LRINCONM\MINSALUD 2012\ORFEO 2019\Usuarios Externos

Bogotá D.C.,

Doctor
GIANCARLO BUITRAGO
Profesor asociado Departamento de Cirugía
Instituto de Investigaciones Clínicas
Universidad Nacional de Colombia
gbuitragog@unal.edu.co
No registra
Bogotá D.C.

ASUNTO: Solicitud información salud, Rad.201842301411402

Cordial saludo.

Atendiendo a su solicitud de registros anonimizados relacionados con las fuentes de información de: Afiliados a Salud, nacimientos y defunciones e Información de Servicios de Salud – Estudio de Suficiencia de 2017 y dando alcance al oficio de respuesta con radicado 201913000267461, nos permitimos informarles que se encuentra disponible el conjunto de datos que se relacionan en la Tabla 1, los cuales fueron copiados en el dispositivo externo suministrado por ustedes.

Tabla 1 Archivos entregados (carpeta:MSyPS-SISPRO-SGD)

Nombre archivo	Número de registros	Descripción
Est_Suf_2017_I.zip	120.310.412	Servicios de salud y medicamentos reportados para el período enero-abril de 2017
Est_Suf_2017_I.zip	127.864.390	Servicios de salud y medicamentos reportados para el período mayo-agosto de 2017
Est_Suf_2017_I.zip	124.769.891	Servicios de salud y medicamentos reportados para el período septiembre-diciembre de 2017
ExtraccionAfilaiciones2017_2018.zip	104.982.027	Datos de las afiliaciones a salud con corte a junio de 2017 y 2018
ExtraccionDefuncionesND 2017_2018_2019	574.505	Defunciones registradas en RUAF-ND para el periodo 2017-01 a 2019-02. Se entrega el archivo con el PersonaID (Identificador único para el fallecido), MadreID (Identificador único para la madre del fallecido en muertes fetales y en menores de 1 año).
ExtraccionNacimientosND 2017_2018_2019	1.328.771	Nacimientos registrados en RUAF-ND para el periodo 2017-01 a 2019-02. Se entrega el

Nombre archivo	Número de registros	Descripción
		archivo con el PersonaID (Identificador único para el fallecido), MadreID (Identificador único para la madre del fallecido en muertes fetales y en menores de 1 año).

Fuente: Elaborado Grupo SGD - OTIC

La información está dispuesta en archivos comprimidos con una contraseña, se solicita enviar notificación de recibido desde el correo autorizado por ustedes al correo electrónico lrincon@minsalud.gov.co para enviar la clave correspondiente; el nombre del asunto deberá ser "Solicitud Clave radicado 201913000349201" de acuerdo a lo establecido en el procedimiento para este fin.

Por parte de esta Oficina se solicita socializar la existencia de estos datos al interior de la Universidad, en aras a facilitar su consulta por parte de los interesados y evitar reprocesos a nivel del Ministerio de esta misma información, optimizando de esta manera los recursos.

Se hace entrega de esta información en los términos de la Ley 1581 de 2012 – *por el cual se dictan disposiciones generales para la protección de datos personales* – y su uso debe garantizar el derecho de habeas data.

Finalmente, es importante resaltar que para el Ministerio de Salud y Protección Social y en particular para esta Oficina será de gran beneficio conocer los resultados de los proyectos de investigación que ustedes adelanten utilizando los datos entregados, por lo que estaremos atentos a conocer el resultado de esos proyectos.

Atentamente,

DOLLY ESPERANZA OVALLE CARRANZA
Jefe Oficina de Tecnología de la Información y la Comunicación

Elaboró: LuzR
Revisó/Aprobó: MaríaE

C:\LRincon\Trabajo LRINCONM\MINSALUD 2012\ORFEO 2017\Usuarios Externos

Bogotá D.C.,

Doctor
GIANCARLO BUITRAGO
Profesor asociado Departamento de Cirugía
Instituto de Investigaciones Clínicas
Universidad Nacional de Colombia
gbuitragog@unal.edu.co
No registra
Bogotá D.C.

ASUNTO: Solicitud información salud, Rad.201842301411402

Cordial saludo.

Atendiendo a su solicitud de registros anonimizados relacionados con las fuentes de información de: Registro Individual de Prestación de Servicios de Salud - RIPS, nacimientos y defunciones y dando alcance al oficio de respuesta con radicado 201913000267461, nos permitimos informarles que se encuentra disponible el conjunto de datos que se relacionan en la Tabla 1, los cuales fueron copiados en el dispositivo externo suministrado por ustedes.

Tabla 1 Archivos entregados (carpeta:MSyPS-SISPRO-SGD)

Nombre archivo	Número de registros	Descripción
RIPS_UNAL_2011	187.799.363	Datos anonimizados de las prestaciones de servicios de salud – RIPS año 2011
RIPS_UNAL_2012	226.655.031	Datos anonimizados de las prestaciones de servicios de salud – RIPS año 2012
RIPS_UNAL_2013	206.980.922	Datos anonimizados de las prestaciones de servicios de salud – RIPS año 2013
RIPS_UNAL_2014	288.816.837	Datos anonimizados de las prestaciones de servicios de salud – RIPS año 2014
RIPS_UNAL_2015	288.537.344	Datos anonimizados de las prestaciones de servicios de salud – RIPS año 2015
RIPS_UNAL_2016	207.392.562	Datos anonimizados de las prestaciones de servicios de salud – RIPS año 2016

Nombre archivo	Número de registros	Descripción
RIPS_UNAL_2017	280.398.850	Datos anonimizados de las prestaciones de servicios de salud – RIPS año 2017
RIPS_UNAL_2018	378.305.880	Datos anonimizados de las prestaciones de servicios de salud – RIPS año 2018
PILA_UNAL_2017	142.605.356	Datos anonimizados de cotizantes año 2017 según estructura de entregas anteriores
ExtraccionNacimientosND2011_2016	3.653.372	Nacimientos registrados en RUAF-ND para el periodo 2011-01 a 2016-12. Se entrega el archivo con el PersonaID (Identificador único para la madre del nacido)
ExtraccionDefuncionesND2011_2016	1.275.657	Defunciones registradas en RUAF-ND para el periodo 2011-01 a 2016-12. Se entrega el archivo con el PersonaID (Identificador único para el fallecido), MadreID (Identificador único para la madre del fallecido en muertes fetales y en menores de 1 año).

Fuente: Elaborado Grupo SGD - OTIC

La información está dispuesta en archivos comprimidos con una contraseña en el dispositivo dispuesto por ustedes. Se solicita enviar notificación de recibido desde el correo autorizado por ustedes al correo electrónico lrincon@minsalud.gov.co para enviar la clave correspondiente; el nombre del asunto deberá ser "Solicitud Clave radicado 201913000640321" de acuerdo a lo establecido en el procedimiento para este fin.

Por parte de esta Oficina se reitera en la solicitud de socializar la existencia de estos datos al interior de la Universidad, en aras a facilitar su consulta por parte de los interesados y evitar reprocesos a nivel del Ministerio de esta misma información, optimizando de esta manera los recursos.

Se hace entrega de esta información en los términos de la Ley 1581 de 2012 – *por el cual se dictan disposiciones generales para la protección de datos personales* – y su uso debe garantizar el derecho de habeas data.

La salud es de todos Minsalud

Al contestar por favor cite estos datos:
Radicado No.: 201913000640321
Fecha: 27-05-2019

Finalmente, tal y como se ha manifestado en otras oportunidades, para el Ministerio de Salud y Protección Social y en particular para esta Oficina será de gran beneficio conocer los resultados de los proyectos de investigación que ustedes adelanten utilizando los datos entregados, por lo que estaremos atentos a conocer el resultado de esos proyectos.

Atentamente,

DOLLY ESPERANZA OVALLE CARRANZA
Jefe Oficina de Tecnología de la Información y la Comunicación

Elaboró: LuzR
Revisó/Aprobó: MaríaE

C:\LRincon\Trabajo LRINCONM\MINSALUD 2012\ORFEO 2018\Usuarios Externos \UNal
