# Supplementary material for: Association of healthcare fragmentation with three-year survival among kidney transplant recipients in Colombia
Source: PLoS One. 2025 Aug 18;20(8):e0316418. doi: 10.1371/journal.pone.0316418 (PMC12360509; doi:10.1371/journal.pone.0316418)
Supplement: Attachment — Submitted filename: Response to Reviewers.docx [file pone.0316418.s003.docx]

**Response to reviewers**

**Oriana Rivera-Lozada de Bonilla
Academic Editor
PLOS ONE**

**Manuscript Title:** Association of healthcare fragmentation and overall survival in patients with kidney transplant in Colombia
**Manuscript ID:** PONE-D-24-57142
**Corresponding Author:** Giancarlo Buitrago

Dear Editors and Reviewers,

We sincerely thank you for the valuable feedback provided on our manuscript. Your thoughtful comments and suggestions have enhanced the work's clarity, rigour, and overall quality. Below, we address each comment individually, providing detailed explanations and outlining the specific changes made to the manuscript.

**Editor comments**

We sincerely thank the Editor for the comments and suggestions provided. In response, we have revised the manuscript to ensure it adheres fully to PLOS ONE’s formatting and style requirements. Specifically, we have ensured that the ethics statement includes all necessary details regarding data use and approvals (Supplementary file 1). In addition, all funding information has been removed from the main text and appropriately included in the cover letter under the “Role of the Funder” section.

With respect to Figure 2, we would like to clarify that it was generated using coordinate data provided by the Colombian National Statistics Department (Departamento Administrativo Nacional de Estadística – DANE). No copyrighted maps or satellite images created using proprietary sources were employed in the figure. Therefore, we respectfully suggest that copyright permissions are not required. We are grateful for all the recommendations, which have helped improve the quality and compliance of the manuscript.

**Reviewer #1:**

**Comment No. 1:** Make the title more engaging and reflective of the results. For example: “Impact of Healthcare Fragmentation on Three-Year Survival of Kidney Transplant Recipients in Colombia.”

**Answer No. 1:** Many thanks for your comment related to the title, we agree with the need for a more informative and engaging title and have adapted it as your suggestions. However, since our study was observational and did not use formal causal inference methods, we believe that the term “impact” may imply causality, which could be misleading. Therefore, we have revised the title to: “Association of Healthcare Fragmentation with Three-Year Survival among Kidney Transplant Recipients in Colombia.”

**Comment No. 2:** The abstract does not mention why healthcare fragmentation is a significant issue in Colombia or LMICs. Add a sentence explaining why fragmentation is relevant in Colombia or LMICs.

**Answer No. 2:** Thank you for this valuable suggestion. We have revised the abstract to include a sentence that contextualizes the relevance of healthcare fragmentation in Colombia and other low- and middle-income countries (LMICs). The revised section now reads:
“Healthcare fragmentation, defined by inadequate communication and lack of integration among providers, disrupts the continuum of care, leading to adverse clinical outcomes. Latin American countries face significant challenges in delivering integrated healthcare. This fragmentation is further exacerbated in Colombia by the decentralized structure of the healthcare system, which disperses responsibilities across multiple actors, including public and private providers, insurers, and regional authorities.”

**Comment No. 3:** The conclusion is clear but repetitive with the introduction. Streamline the conclusion to avoid repetition. Focus on the implications and recommendations.

**Answer No. 3:** We appreciate this observation. To avoid redundancy and enhance the clarity of our conclusions, we have revised the final sentence of the abstract. The updated version reads:
“These findings underscore the importance of integrated care models and improved coordination among providers to enhance patient outcomes, particularly in resource-limited settings.” This streamlined conclusion highlights the study’s implications while minimizing overlap with the introduction.

**Comment No. 4:** While the introduction mentions healthcare fragmentation in general, it does not provide enough context about how fragmentation specifically impacts kidney transplant patients in Colombia. Expand on why Colombia is a relevant setting for studying fragmentation and how its healthcare system's structure creates challenges.

**Answer No. 4:** Thank you for this insightful comment. We agree that it is important to contextualize how healthcare fragmentation specifically affects kidney transplant recipients in Colombia. To address this, we have expanded the introduction to include the following explanation: “This fragmentation is further exacerbated in Colombia by the decentralized structure of the healthcare system, which disperses responsibility across multiple actors, including public and private providers, insurers, and regional authorities. For complex medical procedures such as kidney transplantation, once the surgery is completed, the insurer determines which providers will deliver follow-up care, including medical appointments, laboratory tests and follow-up services. Some insurers maintain agreements with specialized centers, allowing for care within a single institution during the first 6 to 12 months post-transplant. Others, however, lack such arrangements, resulting in patients receiving care from multiple providers. In these cases, the consequences of fragmentation are particularly severe as they encounter obstacles such as inconsistent follow-up, and insufficient communication between transplant centres and other providers. These challenges not only hinder recovery but also increase the likelihood of complications, hospital readmissions, and preventable mortality”

**Comment No. 5:** The phrase "fragmentation manifests both horizontally... and vertically" could be explained more clearly for a general audience.

**Answer No. 5:** Thank you for highlighting this opportunity to improve clarity. We have revised the sentence for broader accessibility, as follows:
“In Colombia, fragmentation manifests as a lack of coordination both across providers at the same level of care (horizontal fragmentation) and between different levels of care and administrative entities such as insurers and public institutions (vertical fragmentation).”

**Comment No. 6:** The definition of healthcare fragmentation ("measured by the number of different healthcare providers") is too simplistic and lacks methodological depth. Provide more detail on how fragmentation was quantified.

**Answer No. 6:** Thank you for this important observation. We have revised the Methods section to provide a more detailed explanation of how healthcare fragmentation was measured. The updated text now reads: **“To measure healthcare fragmentation, we counted the number of unique healthcare providers involved in a patient's care during the first year following kidney transplantation. This approach, used in prior research, serves as a proxy for fragmented care by reflecting the dispersion of services across different providers. Each provider code corresponds to a distinct institution or healthcare facility as recorded in the administrative claims database. While this metric does not capture coordination quality directly, higher counts are assumed to indicate lower care integration.”**

**Comment No. 7:** The rationale for selecting the 75th percentile as the cut-off for high fragmentation is not explained. Justify the use of the 75th percentile for defining high fragmentation.

**Answer No. 7:** Thank you for pointing out the need to clarify this methodological decision. We have now included the following justification in the manuscript:
“We defined high fragmentation as having 11 or more unique providers during the first year post-transplant, corresponding to the 75th percentile of the fragmentation distribution. This threshold was chosen to identify individuals exposed to a substantially higher-than-average degree of fragmentation. Similar percentile-based approaches have been used in health services research to define extreme exposure groups.”

**Comment No. 8:** Descriptions of geographical fragmentation are overly detailed, making it difficult to identify key findings. Focus on the most important findings and present them concisely. Focus on the most important findings and present them concisely. For example: "Patients with high fragmentation (≥11 providers) had a significantly higher mortality rate (18%) compared to those with low fragmentation (12%) (p=0.04)."

**Answer No. 8:** Thank you for your suggestion. To enhance clarity and conciseness, we have summary the geographical fragmentation results and we focused the narrative on key results related to mortality.

**Comment No. 9:** Some results, such as "high fragmentation was associated with a 49% increased mortality risk," are presented without interpretation or context. Briefly explain the implications of key results, e.g.: "This suggests that greater provider involvement, without effective care coordination, negatively impacts patient outcomes."

**Answer No. 9:** Thank you for your observation. We agree on the importance of contextualizing our findings. To address this, we expanded the discussion to include the following interpretation:
“This study evaluated the association between healthcare fragmentation during the first year after kidney transplantation and survival outcomes. Our findings demonstrate that high fragmentation, defined as exposure to 11 or more unique healthcare providers within the first-year post-transplant, was associated with a 49% increased mortality risk within three years (HR: 1.49; 95% CI 1.12–1.97; p=0.01) for each additional provider participating in healthcare during the first-year post-transplant. This suggests that a greater number of providers, in the absence of effective care coordination, may compromise continuity of care and adversely impact patient outcomes.”

**Comment No. 10:** Some terms (e.g., "fragmentación") appear in Spanish (Table 1), which is inconsistent with the rest of the English manuscript. Standardize the language to ensure all terms are in English.

**Answer No. 10:** Thank you for pointing this out. We have carefully reviewed and corrected all language inconsistencies in the manuscript. Specifically, in Table 1, the term “fragmentación” has been replaced with “fragmentation,” and we ensured that all terminology is now presented consistently in English throughout the text.

**Comment No. 11:** Some statements, such as "fragmentation leads to poor outcomes," are too broad and not linked directly to the study findings. Link findings more explicitly to practical implications.

**Answer No. 11:** Thank you for this observation. We revised the relevant section in the discussion to enhance specificity and align it more closely with our findings. We now state:
“Our findings are consistent with the existing literature, which also reports lower survival rates and higher healthcare costs among patients exposed to higher levels of fragmentation.”

**Comment No. 12:** The discussion does not sufficiently address how the findings can be used to inform healthcare policies or improve care coordination in Colombia. Discuss how the findings align or differ from those in other countries to emphasize the study's contribution to global literature.

**Answer No. 12:** Thank you for pointing out this important aspect. In response, we have added a section discussing the implications for healthcare policy and how our findings fit within the international evidence base. The revised text reads: “These findings have direct implications for health policy in countries with fragmented healthcare delivery systems, such as Colombia. Our results underscore the urgent need to implement coordinated care models and standardized referral pathways for kidney transplant recipients. In Colombia, since 2015, national efforts have been underway to standardize the management of priority health conditions through the implementation of Comprehensive Healthcare Service Pathways (Rutas Integrales de Atención en Salud, RIAS). These pathways aim to improve care coordination and ensure continuity across different levels of care. Our study contributes novel evidence from a middle-income country and highlights the need to adapt and extend integration strategies such as RIAS to complex, high-risk populations like kidney transplant recipients.”

**Comment No. 13:** The study could benefit from comparing findings with fragmentation studies in high-income countries to provide a broader context. Avoid overly general statements by linking each claim to specific results from the study.

**Answer No. 13:** To our knowledge, this is the first study to examine the association between healthcare fragmentation and three-year survival among kidney transplant recipients in both low- and high-income country settings. Previous studies in transplant populations have primarily focused on the impact of appointment adherence on graft function and patient survival. However, to date, no research has evaluated care from a healthcare system perspective, specifically addressing fragmentation as done in our study. While healthcare fragmentation has been explored in other chronic conditions, such as liver transplantation and cancer, our findings are consistent with prior literature showing that higher fragmentation is associated with lower survival rates and increased healthcare costs. Mechanisms driving these associations likely include delays in treatment, duplication of medical services, and poor coordination among healthcare providers, all of which undermine the potential benefits of transplantation.”

**Comment No. 14:** The conclusion restates key findings but could be more impactful. Focus on actionable recommendations for reducing fragmentation and improving patient outcomes. For example: "Our findings highlight the urgent need to implement integrated care models and enhance care coordination to reduce mortality risks in kidney transplant patients. Policymakers should prioritise strategies to streamline healthcare delivery, particularly in fragmented systems like Colombia."

**Answer No. 14:** Thank you for the suggestion. We have revised the conclusion to emphasize concrete recommendations and future research priorities. The updated conclusion now reads: “Our findings emphasise the importance of integrated healthcare models and enhance care coordination to reduce fragmentation, mortality risks, and optimise resource use in kidney transplant patients. Policymakers should prioritise strategies to streamline healthcare delivery, particularly in resource-limited settings and fragmented systems like Colombia. Further research is needed to explore effective strategies for integrating care and addressing regional disparities in healthcare access, which could inform policies to improve outcomes for patients with chronic conditions, including those requiring renal transplantation.”

**Reviewer #2:**

I appreciate your hard work.

**Comment No. 1:** In the "Financial Disclosure" section of PLOS One Submission System, part please add where the partial funders played specific roles, like- Study design, Data collection and analysis, Decision to publish or Preparing the manuscript. If the funders have no role you can simply write it as, "The funders did not contribute in study design, fieldwork, data analysis, decision to publish or preparation of the manuscript." But if they do, please mention in which specific sector they contributed.

**Answer No. 1:** Thank you for your guidance. We have revised the financial disclosure section in the submission system to specify the role of the funders. As the funders had no involvement in the study design, data collection and analysis, decision to publish, or manuscript preparation, the updated statement now reads:
“The funders did not contribute to the study design, fieldwork, data analysis, decision to publish, or preparation of the manuscript.”

**Comment No. 2:** As per your statement, "To our knowledge, no studies have specifically evaluated the association between healthcare fragmentation and survival outcomes in renal transplant recipients in low- and middle-income countries (LMICs)." So, while you have mentioned that we don't have data in LMICs, but is there any in HICs? please look for the available studies on similar studies conducted in High Income Countries so that a contrast can be build up in Discussion section. If there is no studies available in HICs you can mention it. That will give stronger rationale for the study.

**Answer No. 2:** Thank you for this important observation. In response, we conducted a comprehensive review of the literature and identified that while studies in high-income countries have examined factors such as appointment adherence, and clinical follow-up in kidney transplant outcomes, none have specifically analyzed healthcare fragmentation at the health system level as a determinant of post-transplant survival. We have revised the discussion to state: “To our knowledge, this is the first study to examine the association between healthcare fragmentation and three-year survival among kidney transplant recipients in both low- and high-income country settings. Previous studies in transplant populations have primarily focused on the impact of appointment adherence on graft function and patient survival. However, to date, no research has evaluated care from a healthcare system perspective, specifically addressing fragmentation as done in our study. While healthcare fragmentation has been explored in other chronic conditions, such as liver transplantation and cancer, our findings are consistent with prior literature showing that higher fragmentation is associated with lower survival rates and increased healthcare costs.”

**Comment No. 3:** Deeper analysis and findings are illustrated with well structured and readable figures and tables.

**Comment No. 4:** As mentioned, please find some existing studies conducted in High Income Setting so that this study can compare and contrast key features and increase its strength.

**Answer No. 4:** Thank you for reiterating this important point. As detailed in our response to Comment No. 2, we reviewed relevant literature from high-income countries. While previous studies have examined clinical determinants of transplant success such as follow-up attendance, they have not explored structural healthcare fragmentation. We have updated the discussion to reflect this contrast and emphasize how our study adds new insights by evaluating system-level fragmentation, thus contributing evidence from an LMIC perspective to an underexplored dimension of transplant care research.

**Comment No. 5:** Kindly remove the funding source in the "Acknowledgments" or anywhere else in the manuscript file. Funding information should only be entered in the "financial disclosure" section of the submission system as mentioned earlier in the review.

**Answer No. 5:** Thank you for the clarification. We have removed all mentions of the funding source from the “Acknowledgments” section and the manuscript file, in compliance with PLOS ONE guidelines. The funding details are now exclusively stated in the “Financial Disclosure” section of the submission system.

**Reviewer #3:**

**Comment No. 1:** The introduction provides context on healthcare fragmentation and why it is an important issue.

**Answer No. 1:** Thank you for your positive feedback. We are glad that the introduction successfully conveys the relevance of healthcare fragmentation as a public health issue and sets the stage for the study.

**Comment No. 2:** The literature review is relevant and establishes the need for the study.

**Answer No. 2:** Thank you for this comment. We appreciate your recognition of the literature review’s contribution to justifying the study’s objectives and contextualizing the research question.

**Comment No. 3:** The study design is appropriate for the research question.

**Answer No. 3:** Thank you for your assessment. We agree that a retrospective cohort design was suitable for evaluating the association between healthcare fragmentation and survival in kidney transplant recipients using administrative health data

**Comment No. 4:** Ethical considerations are mentioned (if applicable).

**Answer No. 4:** Thank you for your comment. We confirm that all ethical approvals were obtained in accordance with national regulations, and we have ensured this information is clearly stated in the manuscript.

**Comment No. 5:** The results are well-structured and follow a logical order.

**Answer No. 5:** We appreciate your positive evaluation. We aimed to present the results in a clear and logical sequence, supported by appropriately formatted tables and figures.

**Comment No. 6:** Use of tables and figures helps in presenting data effectively.

**Answer No. 6:** Thank you for your feedback. We are pleased to know that the tables and figures were effective in enhancing the presentation and interpretation of our findings.

**Comment No. 7:** The discussion links findings to the broader literature.

**Answer No. 7:** Thank you for this observation. We aimed to situate our findings within the global body of literature on care fragmentation and transplant outcomes, and have further expanded the discussion to include comparisons with studies from high-income countries.

**Comment No. 8:** The practical implications of the results are explored.

**Answer No. 8:** We appreciate this comment. In response to suggestions from multiple reviewers, we strengthened the discussion and conclusion to more explicitly highlight actionable implications for healthcare policy and practice, particularly in the context of LMICs like Colombia.

**Comment No. 9:** Strengths: Summarizes key findings effectively.

**Answer No. 9:** Thank you for acknowledging the strengths of our conclusion. We have further revised this section to make it more impactful and policy-relevant, while ensuring it remains a concise summary of our key findings.

**Reviewer #4:**

This study explores the link between healthcare fragmentation during the first year after kidney transplantation and three-year mortality risk in Colombia. The analysis of national administrative data revealed that higher fragmentation significantly increases mortality risk, even when adjusting for age, gender, region, comorbidities, and transplant year, with a 49% higher risk for those experiencing high fragmentation.
Building on the teams' prior research, this study highlights the importance of care coordination for chronic disease patients. Its findings are especially relevant for low- and middle-income countries (LMICs), where healthcare systems tend to be fragmented and under-resourced.

Strengths:
The manuscript is clearly structured and written with clarity.
The use of a national database enhances the study’s generalizability and policy relevance.
The findings are communicated in an accessible manner, with appropriate use of tables and figures.

Weaknesses:
The arguments could be better situated within the existing literature, especially regarding kidney transplantation and healthcare fragmentation in comparable contexts.
Overall Recommendation:
This is a well-written and policy-relevant manuscript addressing a significant public health issue. I recommend acceptance pending revision of the points below.

Major Issues
Methods

**Comment No. 1:** Lines 148–150- The authors should elaborate on how fragmentation scores were derived from the database. Were they based on the specific services provided? How are services that require multiple health workers accounted for? Although the authors have referenced previous studies that used this measure, further information is necessary to clarify its validity for their current research.

**Answer No. 1:** Thank you for this important observation. We agree that further clarification was necessary and have expanded the corresponding section of the Methods. Given the high prevalence of multimorbidity among kidney transplant recipients, such as as hypertension, diabetes mellitus, metabolic syndrome, among others, we considered essential to include all types of healthcare services received, not only those directly related to transplantation. Comprehensive post-transplant care is critical, as favorable outcomes depend on the integration of multiple services. For this reason, we have revised the Methods section as follows:

“We included all types of services, beyond transplant-specific care, to capture the full scope of medical attention required for these patients. This approach reflects the complexity of managing comorbidities and the need for integrated care.”

**Comment No. 2:** Lines 154–155- More detail is needed regarding Colombia’s insurance system and how the geographic regions were categorized. Also more information on the Charlson Comorbidity Index (CCI) is important for the general audience, a brief description of how it is calculated, what conditions it includes, and its relevance to transplant populations would enhance clarity, especially for readers outside the clinical field.

**Answer No. 2:** Thank you for this comprehensive suggestion. We have implemented the following clarifications:

- Colombia’s insurance system: A description was added: “Colombia, despite achieving universal health coverage through contributory (formal employees and their families) and subsidised (informal workers and their families) schemes covering over 97% of the population, faces persistent challenges related to healthcare fragmentation.”
- Geographic regions: Detailed descriptions of regions were added to Table 1 and 2 footnotes.
- Charlson Comorbidity Index: We included the following explanation: “The Charlson Comorbidity Index (CCI) was validated for use in Colombia by Oliveros et al. derived from ICD-10 codes. The CCI considered the presence of the following conditions: acute myocardial infarction, congestive heart failure, peripheral vascular disease, stroke, dementia, chronic pulmonary disease, connective tissue disease, peptic ulcer disease, liver disease, diabetes, complications of diabetes, cancer, metastatic cancer, paraplegia, renal disease, severe liver disease, and HIV.”

**Comment No. 3:** Table 2- Each region should have its own unique subscript, such as "a," "b," and "c," with explanations in the footnotes as before. Bogotá, D.C. should be labeled as "Metropolitan" if more than one region is combined here (it seems so).

**Answer No. 3:** Thank you for the helpful suggestion. Table 2 has been revised with unique subscripts and clearly defined footnotes. The updated regional classifications are:
^a^ Atlántica: Atlántico, Bolívar, Cesar, Córdoba, Magdalena, La Guajira, Sucre, and San Andrés;

^b^ Metropolitan: Bogotá D.C. and Cundinamarca;

^c^ Central: Antioquia, Caldas, Huila, Quindío, Risaralda, and Tolima;

^d^ Oriental: Arauca, Boyacá, Caquetá, Casanare, Meta, Norte de Santander, Santander, and Vichada;

^e^ Pacífica: Cauca, Chocó, Nariño, Putumayo, and Valle del Cauca;

^f^ Orinoquía and Amazonía: Amazonas, Guainía, Guaviare, and Vaupés.

**Comment No. 4:** Line 261–267- The authors compare their findings to research on other chronic conditions. However, the management and care coordination needs of kidney transplant patients differ substantially from those with cancers for example. The argument would be more persuasive if supported by literature on kidney transplantation, As the authors mentioned that their work is the first in LMICs, it is important to consider findings related to kidney transplants from non-LMICs. Comparing their results with studies specifically focused on kidney transplants could provide valuable context. The cited study may not be sufficient to align their findings with existing literature. This is a crucial point that needs to be addressed.

**Answer No. 4:** Thank you for this important recommendation. We agree that kidney transplant care differs significantly from that of other chronic diseases and have revised the discussion to address this. Specifically, we now state: **“Previous studies in transplant populations have primarily focused on the impact of appointment adherence on graft function and patient survival. However, to date, no research has evaluated care from a healthcare system perspective, specifically addressing fragmentation as done in our study. While healthcare fragmentation has been explored in other chronic conditions, such as liver transplantation and cancer, our findings are consistent with prior literature showing that higher fragmentation is associated with lower survival rates and increased healthcare costs.”**

**Comment No. 5:** Line 267–270- The manuscript references general issues with fragmentation in other countries but lacks detail about Colombia’s healthcare system. Are delays in treatment, duplicative services, and poor care coordination documented problems locally?

**Answer No. 5:** Thank you for this comment. We have expanded the manuscript to document Colombia-specific challenges. The revised discussion includes: **“For complex medical procedures such as kidney transplantation, once the surgery is completed, the insurer determines which providers will deliver follow-up care, including medical appointments, laboratory tests and follow-up services. Some insurers maintain agreements with specialized centers, allowing for care within a single institution during the first 6 to 12 months post-transplant. Others, however, lack such arrangements, resulting in patients receiving care from multiple providers. In these cases, the consequences of fragmentation are particularly severe as they encounter obstacles such as inconsistent follow-up, and insufficient communication between transplant centres and other providers. These challenges not only hinder recovery but also increase the likelihood of complications, hospital readmissions, and preventable mortality.”**

**Comment No. 6:** Limitations- The authors noted key limitations in their study related to clinical information important for patient survival. However, they did not specify details that could confound or interact with their results. this information is for the study's conclusion.

**Answer No. 6:** Thank you for pointing this out. We have updated the limitations section in the conclusion to explicitly mention potential unmeasured confounding factors. The revised text reads: **“Administrative databases lack detailed clinical information, which may leave behind some crucial covariables. To mitigate this limitation, we incorporated comorbidity data using the CCI, a validated scale for its use with administrative datasets in Colombia. The selection and interpretation of this index were further supported by clinical experts in transplantation.”**

**Comment No. 7:** References- Reference 13 appears to be already published, and the DOI provided is incorrect. The same article seems to be cited again as Reference 15. Please verify and adjust accordingly.

**Answer No. 7:** Thank you for catching this duplication. We have verified the publication status of Reference 13, corrected its DOI, and removed the redundant citation that appeared as Reference 15. All in-text references have been updated accordingly to maintain accuracy.

**Comment No. 8:** Line 79–81: A citation is needed to support the description of Colombia’s healthcare system.

**Answer No. 8:** Thank you. We have added a relevant citation to support the description of Colombia’s healthcare system, referencing national health system analyses that detail its structure and regulatory framework.

**Comment No. 9:** Lines 81–86: Important claims are made to justify the research question, but no supporting literature is cited.

**Answer No. 9:** Thank you for this observation. We have revised the paragraph and included appropriate references to support the importance of the research question.

**Comment No. 10:** Line 89–90: Citing similar studies from high-income countries would strengthen the rationale for the study.

**Answer No. 10:** We agree and have revised the paragraph to include citations from similar studies in high-income settings that have reported associations between fragmented care, non-adherence, and adverse post-transplant outcomes. The paragraph now reads “To our knowledge, no studies have specifically evaluated the association between healthcare fragmentation and survival outcomes among kidney transplant recipients in either high-income countries (HICs) or low- and middle-income countries (LMICs). Some studies have explored fragmentation in other transplant populations, such as liver transplant recipients, and have reported adverse outcomes, including increased mortality and risk of rehospitalization [18,19]. This gap in the literature highlights the need for focused research on how fragmented care affects kidney transplant outcomes, particularly in settings like Colombia, where systemic fragmentation is a well-documented barrier to effective healthcare delivery.”

**Comment No. 11:** Line 104–106: It is unclear whether the study truly addresses all the claims made in this paragraph.

**Answer No. 11:** Thank you for this critical remark. We have reviewed and revised the paragraph to ensure it accurately reflects the scope and focus of our study. The text now reads “This study aims to address this gap by assessing the association of care fragmentation experienced by number of providers that attended renal transplant patients during the first year following transplantation and the three-year overall survival. The findings can potentially inform health policies and interventions to reduce care fragmentation, improve coordination among healthcare providers, and optimise resource use in this population. Ultimately, the study would highlight the importance of integrated care models in enhancing renal transplant patients' survival, particularly in resource-limited and fragmented healthcare systems like Colombia.”

**Comment No. 12:** Line 169–170: I suggest including the global test p-value from the Schoenfeld residuals.

**Answer No. 12:** Thank you for your suggestion. We have added the global p-value from the Schoenfeld residuals test to the manuscript to confirm that the proportional hazards assumption of the Cox model was not violated. In the manuscript now reads “No violation of the proportional-hazards assumption was detected. The global Schoenfeld test yielded X2 = 16.89 (df =23, p=0.815) and no covariate showed an individual p-value < 0.05.”

**Comment No. 13:** Line 173: Consider adding a STROBE checklist as supplementary material.

**Answer No. 13:** We appreciate the suggestion and have included a completed STROBE checklist as supplementary file 1, in the revised submission to ensure compliance with reporting guidelines.

**Comment No. 14:** Lines 179–181: These participant exclusion criteria should be moved to the Methods section. The rationale for excluding individuals <18 years should be stated. If this is due to different survival patterns, why not also consider an upper age limit (e.g., >75)?

**Answer No. 14:** Thank you for this comment. We have moved the exclusion criteria to the Methods section. Patients under 18 were excluded because pediatric transplant care follows distinct clinical protocols and survival trajectories. We opted not to exclude older adults to reflect real-world heterogeneity and maintain external validity, acknowledging that elderly patients form an important subgroup of transplant recipients.

**Comment No. 15:** Table 1: Clarify “Mean of different healthcare; X (SD)” in a footnote, please also, explain what is meant by “other department” under the region variable.

**Answer No. 15:** Thank you for this observation. We have clarified the column header in Table 1 to read: **“Number of healthcare providers; X (SD)” and added the abbreviation of X: mean in the legend of table 1**. Additionally, we specified “other departments” as: Orinoquía and Amazonía and described the departments within each category in table 1 legend.

**Comment No. 16:** Table 2: Same labeling issues as noted above. Ensure clarity in regional categories.

**Answer No. 16:** Thank you again. As mentioned previously, we revised Table 2 to include unique subscripts for each region and provided clear.

**Comment No. 17:** Table 3 and 4: Statistically significant values should be marked (e.g., using asterisks or bold text) for easier interpretation.

**Answer No. 17:** Thank you for the practical suggestion. We have revised Tables 3 and 4 to highlight statistically significant results using asterisks. A legend was added below the tables to indicate: *: statistically significant (p < 0.05)

**Comment No. 18:** Results – Were there significant differences in unadjusted models? If so, consider including or mentioning these results.

**Answer No. 18:** Thank you for pointing this out. We have added to the text the following information “Table 3 shows the crude three-year mortality proportions for each baseline characteristic evaluated. Patients exposed to low fragmentation had a mortality proportion of 0.12, while those exposed to high fragmentation had a mortality proportion of 0.18 (p=0.04), consistent with the estimates from the unadjusted Cox model (HR: 1.49 (95% IC: 1.17-1.90), p=0.001). Other characteristics where differences were observed included age category and CCI. No differences were found by sex or geographic region.”
